# Prediction of breeding regions for the desert locust *Schistocerca gregaria* in East Africa

**DOI:** 10.1038/s41598-020-68895-2

**Published:** 2020-07-20

**Authors:** Emily Kimathi, Henri E. Z. Tonnang, Sevgan Subramanian, Keith Cressman, Elfatih M. Abdel-Rahman, Mehari Tesfayohannes, Saliou Niassy, Baldwyn Torto, Thomas Dubois, Chrysantus M. Tanga, Menale Kassie, Sunday Ekesi, David Mwangi, Segenet Kelemu

**Affiliations:** 1 International Centre of Insect Physiology and Ecology (ICIPE), P.O. Box 30772-00100, Nairobi, Kenya; 2 Desert Locust Control Organization for Eastern Africa, P.O. Box 30023-00100, Nairobi, Kenya; 3 Plant Protection Services, Ministry of Agriculture, Livestock and Fisheries, P.O. Box 34188-00100, Nairobi, Kenya; 4 Food and Agricultural Organization, Viale delle Terme di Caracalla, 00153 Rome, Italy

**Keywords:** Ecology, Environmental sciences

## Abstract

Desert locust outbreak in East Africa is threatening livelihoods, food security, environment, and economic development in the region. The current magnitude of the desert locust invasion in East Africa is unprecedented and has not been witnessed for more than 70 years. Identifying the potential breeding sites of the pest is essential to carry out cost-effective and timely preventive measures before it inflicts significant damage. We accessed 9,134 desert locust occurrence records and applied a machine-learning algorithm to predict potential desert locust breeding sites in East Africa using key bio-climatic (temperature and rainfall) and edaphic (sand and moisture contents) factors. Ten days greenness maps from February 2020 to April 2020 were overlaid in model outputs to illustrate the temporal evolution of breeding site locations. This study demonstrated that vast areas of Kenya and Sudan, north eastern regions of Uganda, and south eastern and northern regions of South Sudan are at high risk of providing a conducive breeding environment for the desert locust. Our prediction results suggest that there is need to target these high-risk areas and strengthen ground surveillance to manage the pest in a timely, cost-effective, and environmentally friendly manner.

## Introduction

The desert locust *Schistocerca gregaria,* one of about a dozen species of locusts, is a species of swarming short-horned grasshoppers that can migrate great distances during its gregarious phase^1–3^. As they swarm, they voraciously feed on key staple crops such as maize and sorghum, pastures, and any green vegetation that comes their way, thereby significantly affecting the livelihoods of smallholder farmers and pastoralists^4–6^. In Africa, the countries of the Sahel region, especially Algeria, Burkina Faso, Chad, Ethiopia, Eritrea, Mauritania, Mali, Niger, Nigeria, Senegal, Somalia, and Sudan, are particularly susceptible to desert locust outbreaks. Until the 1960s, locust outbreaks frequently occurred, however, post-1960s, outbreaks were less frequent and occurred, on average, only once in a decade^7^.

In general, the desert locust breeds extensively in semi-arid zones extending from West Africa through the Middle East to Southwest Asia, threatening livelihoods of the population in over 65 countries. Between 2019–2020, unprecedented locust breeding was observed in Eritrea, Somalia, and Yemen due to unusually heavy rainfall in the horn of Africa between October to mid-November 2019, more than 400% above average^8^. Following this breeding, countries in the horn of Africa, such as Ethiopia, Kenya, and Somalia, are experiencing extraordinary swarms never witnessed during the past 25 years. The current swarm is estimated to consume ~ 1.8 million MT of vegetation per day across 123,200 km^2^, which represents 11% of Ethiopia's total land area^9^. In Kenya, the locust has spread to approximately 107,000 km^2^ (20% of Kenya) (Kenyan Multi-Agency Team on Desert locust, 2020), and very recently, the locust has invaded Uganda, South Sudan, and Tanzania. It is anticipated to move northward into Sudan and possibly northern part of Chad. The current management strategy of the locust swarm is aerial spraying with chemical pesticides, which has a high cost on humans, livestock, and the environment in addition to its economic burden at the national level biodiversity.

Studies have shown that desert locust has the ability to change its behaviour, ecology, and physiology in response to the changes in climatic conditions^10^. In particular, within a few weeks, swarming adults mature, mate, and begin to oviposit in soils at 10–15 cm below ground in suitable environments in the invaded zones^2,11^. Suitability for oviposition and subsequent breeding is influenced by factors such as soil type, sand content, soil moisture, surface air temperature, rainfall, and prevalence of vegetation^2,12^. The emerging hoppers (nymphs), which are the wingless juvenile stage, can concentrate to become more gregarious and form bands that crawl on the surface over long distances. After several moultings, up to six times, hoppers transition to adults which can come in contact to form a gregarious phase^2^. The time needed for the desert locust to transition from one stage to the other is highly dependent on the weather patterns^13,14^. Both the hopper bands and adult swarms can cause significant damage to the vegetation and crops in the invaded zones. Therefore, to prevent catastrophic swarms from maturing hoppers, it is critical to strengthen ground and aerial surveillance efforts to identify potential breeding sites for timely and effective management of hopper bands. However, effective ground and aerial surveillance are constrained by various factors including extensive area of invasion (e.g., 107,000 km^2^ in Kenya), inaccessibility of invasion zones due to poor infrastructure, limited resources, lack of human capacity for monitoring and control, and difficulties in predicting suitable areas for breeding and outbreaks. Such constraints are typical to the currently invaded zones in Kenya, Uganda, and South Sudan, and to other nearby countries at risk.

Previous desert locust outbreaks in the Horn of Africa were observed in 1996–1998, and it affected countries along the Red Sea, with infestations primarily concentrated in Saudi Arabia and, to a lesser extent, in Egypt, Ethiopia, Eritrea, Northern Somalia, Sudan, and Yemen. Countries such as Kenya and Uganda have not experienced the current level of desert locust invasion for more than 70 years, and little or no information is available on the suitability of specific sites for desert locust oviposition and breeding^13^. Such information is urgently needed to strengthen surveillance (ground and aerial) efforts, regional coordination, and preparedness, inform efforts and improve the delivery of preventive measures before the newly emerging hoppers cause damage.

Locust (desert locust and grasshopper) outbreak prediction and monitoring can be modelled using ecological niches (EN) approaches^15,16^. A category of EN models apply machine learning algorithms that correlate a set of environmental conditions (e.g., bio-climatic variables) to species presence and absence records to predict its suitable habitats^17^. For instance, maximum entropy (MaxEnt), genetic algorithm for rule-set production (GARP), and ecological niche factor analysis (ENFA) are EN tools that predict species suitability using presence-only data^18,19^. MaxEnt was revealed to provide a reasonably better result compared to other presence-only models^18^. In specific, MaxEnt assumes that the suitable areas for occupancy by species would corroborate to the physics principle of maximum entropy without any environmental restrictions. The model predicts habitat suitability by fitting a probability distribution for the incidence of the species across the whole area. However, MaxEnt often experiences overfitting at low threshold levels than, e.g., GARP models^19^.

The objective of this paper is to develop a decision support tool that enables governments and their development partners to control the locust invasion from its breeding sites effectively. The specific objectives are to (1) model the relationship between known desert locust breeding sites around the world with critical bio-climatic (temperature and rainfall) and edaphic (sand and moisture contents) variables using MaxEnt EN model, and (2) validate the model with the existing database, and further develop predictions on potential areas for desert locust oviposition and breeding in Kenya, Uganda, South Sudan, and Sudan.

## Results

### Model validation and evaluation of the performance of the projections to other nations

**Figure 1:**
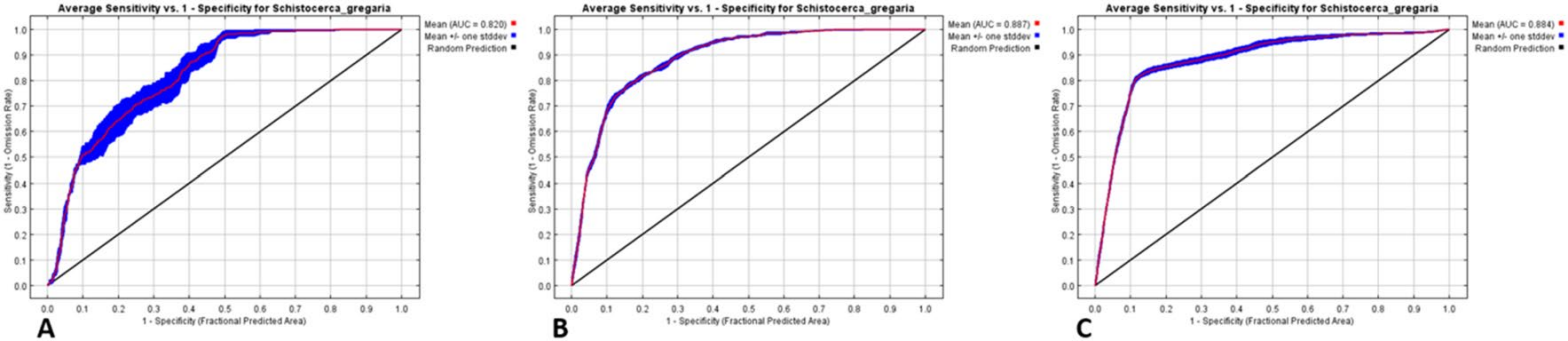
Area under curve for (**A**) Morocco, (**B**) Mauritania, and Saudi Arabia (**C**) models for evaluating the performance of predicting desert locust breeding sites. Zero values for AUC indicate an impossible occurrence area, while values of 1 indicate optimal occurrence area.

The area under the curve (AUC) on the graphs confirmed that all individual models performed well in predicting Morocco (Fig. 1A), Mauritania (Fig. 1B), and Saudi Arabia (Fig. 1C) desert locust breeding areas. The model generated from Mauritania had the highest mean AUC value (0.887), followed by the models from Saudi Arabia (0.884), and Morocco (0.820).

**Figure 2:**
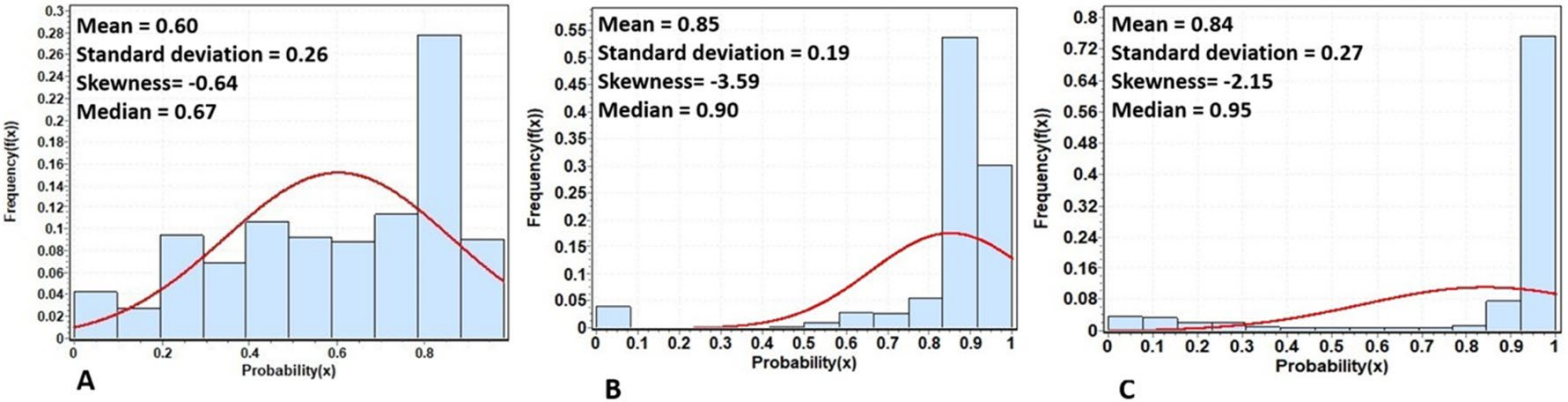
Histogram and normal distribution fit for (**A**) Morocco model, (**B**) Mauritania model projected from Morocco, and (**C**) Saudi Arabia model projected from Morocco.

The results show that the Morocco model parameters obtained from the MaxEnt algorithm (Fig. 2A) performed the best for projecting desert locust breeding sites to other countries as compared to Mauritania and Saudi Arabia (results are not shown) models. In specific, Fig. 2B,C reveal validation of the Morocco model projecting to Mauritania and Saudi Arabia using independent presence records that were not used during model development. The Morocco model projecting to Mauritania had the highest mean of 0.85 with skewness of − 3.59, and a median of 0.9 (Fig. 2B), followed by the Morocco model projecting to Saudi Arabia with a mean of 0.84, a skewness of − 2.15, and a median of 0.95 (Fig. 2C).

**Figure 3:**
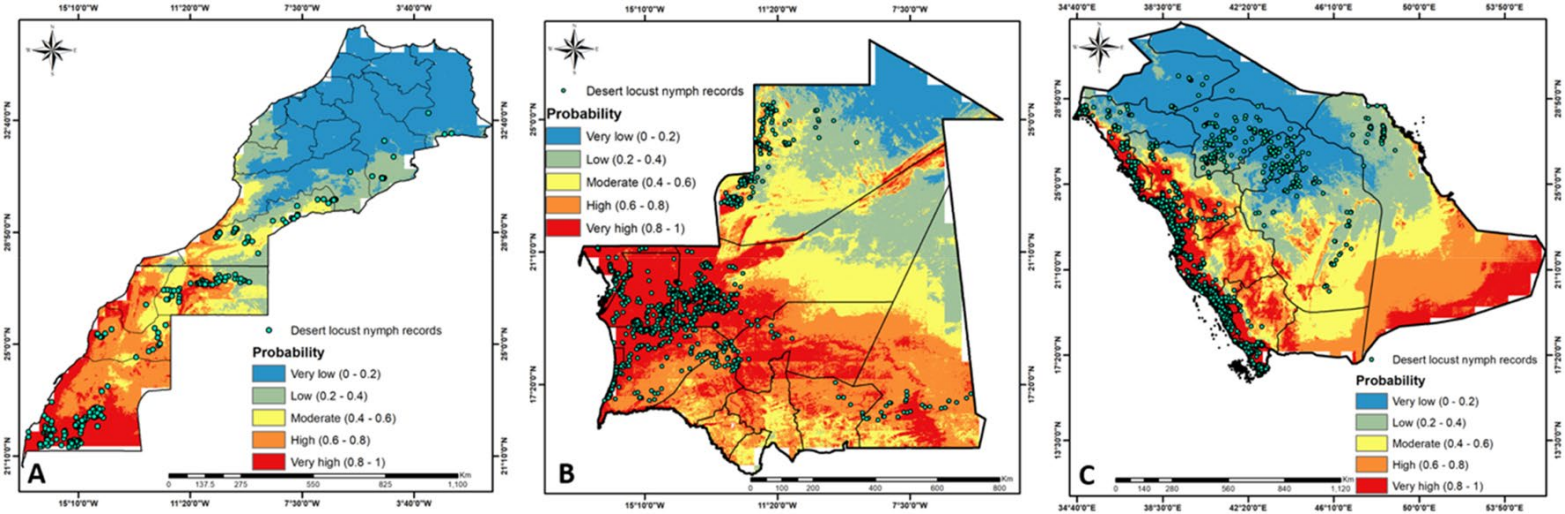
(**A**) Graphical representation of the ecological niche model for breeding sites of desert locusts in Morocco obtained from MaxEnt with Morocco presence records. (**B**) Projected model for Mauritania from Morocco model. (**C**) Projected model for Saudi Arabia from Morocco model. Warmer colors (red) show areas with better-predicted conditions and the green dots indicate the presence locations used for validating the projected models. The figure was generated using the QGIS 3.10.2 software (https://qgis.org/downloads/)^21^.

Moreover, the results reveal that the predicted high and very high suitability scores for desert locust breeding sites in the three countries (i.e., Morocco, Mauritania and Saudi Arabia) matched with known desert locust nymph presence locations (Fig. 3), except in the northeastern Morocco (Fig. 3A), northern Mauritania (Fig. 3B), and towards the central region of Saudi Arabia (Fig. 3C). This confirms the good performance of the Morocco MaxEnt model when projected to the other two countries.

### Environmental variable importance and impact

Table 1 presents the contribution of each environmental variable in the Morocco model projecting to East Africa for predicting desert locust breeding sites. Although rainfall, temperature, soil moisture, and sand content played a considerable role in the model, their level of importance varied. Temperature had the highest contribution in the model, while rainfall had the least contribution (Table 1).

**Table 1:** Percentage contribution of environmental variables in the Morocco model of predicting desert locust breeding sites to East Africa.

Variable	% Contribution
Temperature	70.2
Soil moisture	24.7
Sand content	3.9
Rainfall	1.2

**Figure 4:**
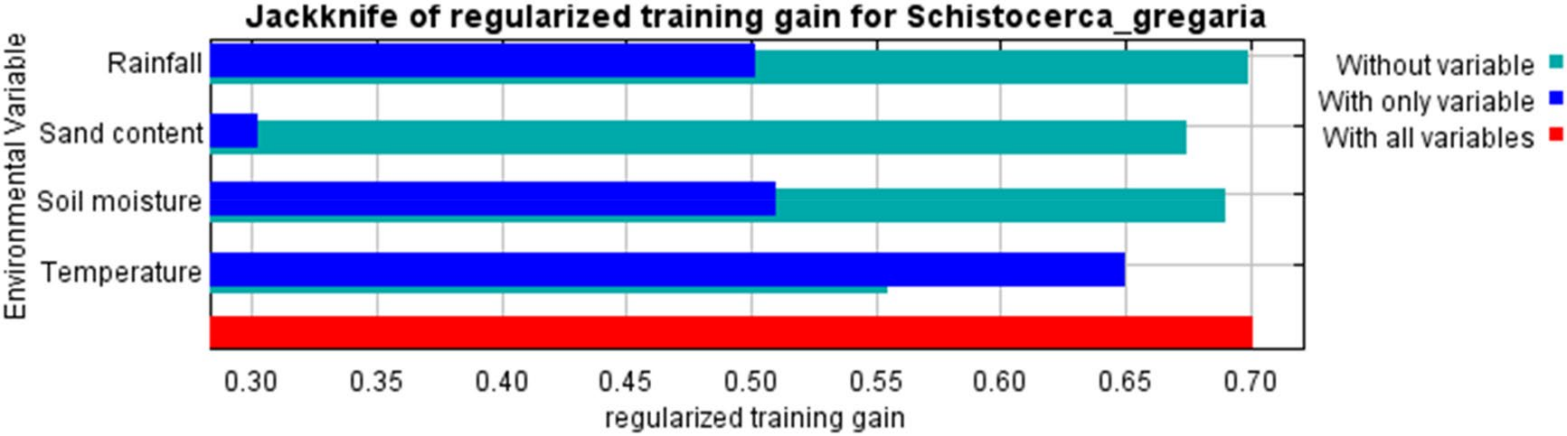
Jackknife of regularized training for Morocco model of desert locusts breeding sites projected to East Africa. Temperature and soil moisture have the highest gain when used in isolation.

Results from the jackknife test of variable importance (Fig. 4) indicate that the variable with the highest gain, when used in isolation, was temperature. This variable decreases the model value of gain the most when it is omitted.

### Modelling desert locust breeding suitability in East Africa

**Figure 5:**
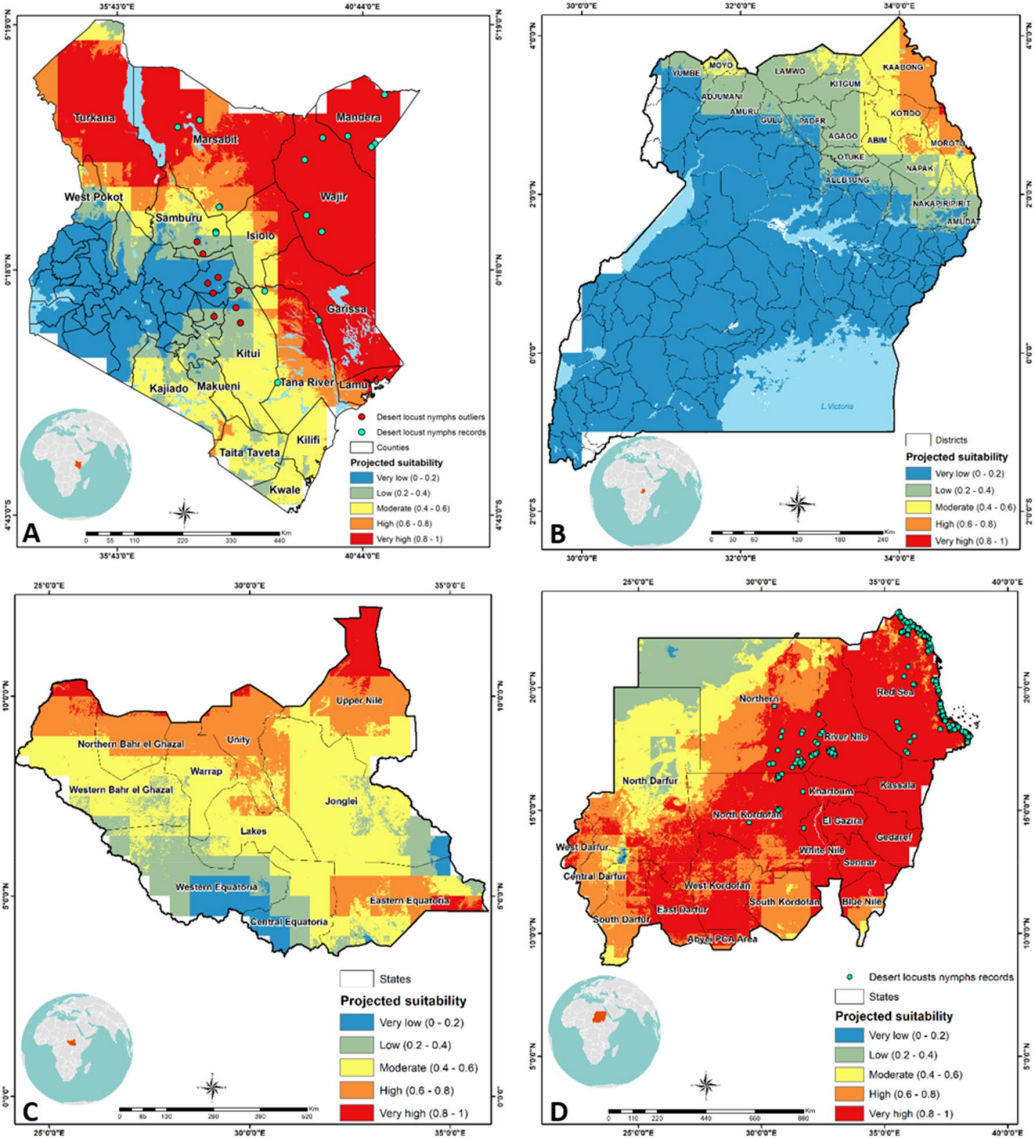
A graphical representation of the projected model for desert locust breeding sites in Kenya (**A**), Uganda (**B**), South Sudan (**C**), and Sudan (**D**) from Morocco model .The dots found in Sudan (647) and Kenya (28) are historical (from 2013 to 2019) and actual (2020) records, respectively, used for measuring the developed model performance. The figure was generated using the QGIS 3.10.2 software (https://qgis.org/downloads/)^21^.

Overall, the results indicate that there is a high probability for desert locust breeding in the northern and eastern regions of Kenya and most parts of Sudan (Fig. 5A,D). However, breeding sites are low in Uganda (Fig. 5B) and restricted to the northern regions bordering South Sudan and Kenya. South Sudan is at risk of breeding in the northern regions and the south east corner bordering Kenya (Fig. 5C).

Based on the established model for Morocco projected in Kenya, it is estimated that there is a high probability for desert locust breeding sites in the northern and eastern counties of Mandera, Wajir, Marsabit, Isiolo, Garissa, and Turkana (Fig. 5A). Few hotspots can also be expected in Samburu, West Pokot and Northern parts of Tana River. Current swarms of desert locust have not moved towards the Kenyan coast. In the event this happens, some few pockets of breeding sites can also be foreseen in Lamu. Other counties in Kenya are mostly unsuitable for breeding and establishment of desert locusts.

The Morocco model projected in Uganda estimated that districts with high probability for desert locust breeding exist in the north, east, and northeast, especially Kotido, Kaabong, and Moroto, followed by Napak, Abim, Kitgum, Moyo, and Lamwo districts (Fig. 5B). Most of the other regions are unsuitable for desert locust breeding. In South Sudan, regions with high probability for the establishment of desert locust breeding sites exist in northern Bahr el Ghazal, Unity, Upper Nile, and Eastern Equatoria, followed by Warrap, Lakes, and some parts of Jonglei (Fig. 5C). Based on the Morocco model, it is estimated that in Sudan, the regions from Darfur in the Southwest to the Red Sea in the northeast, except the northwestern region, have a high probability for the establishment of desert locust breeding sites (Fig. 5D).

### Vegetation impact on desert locust breeding sites in Kenya, Uganda, South Sudan, and Sudan

**Figure 6:**
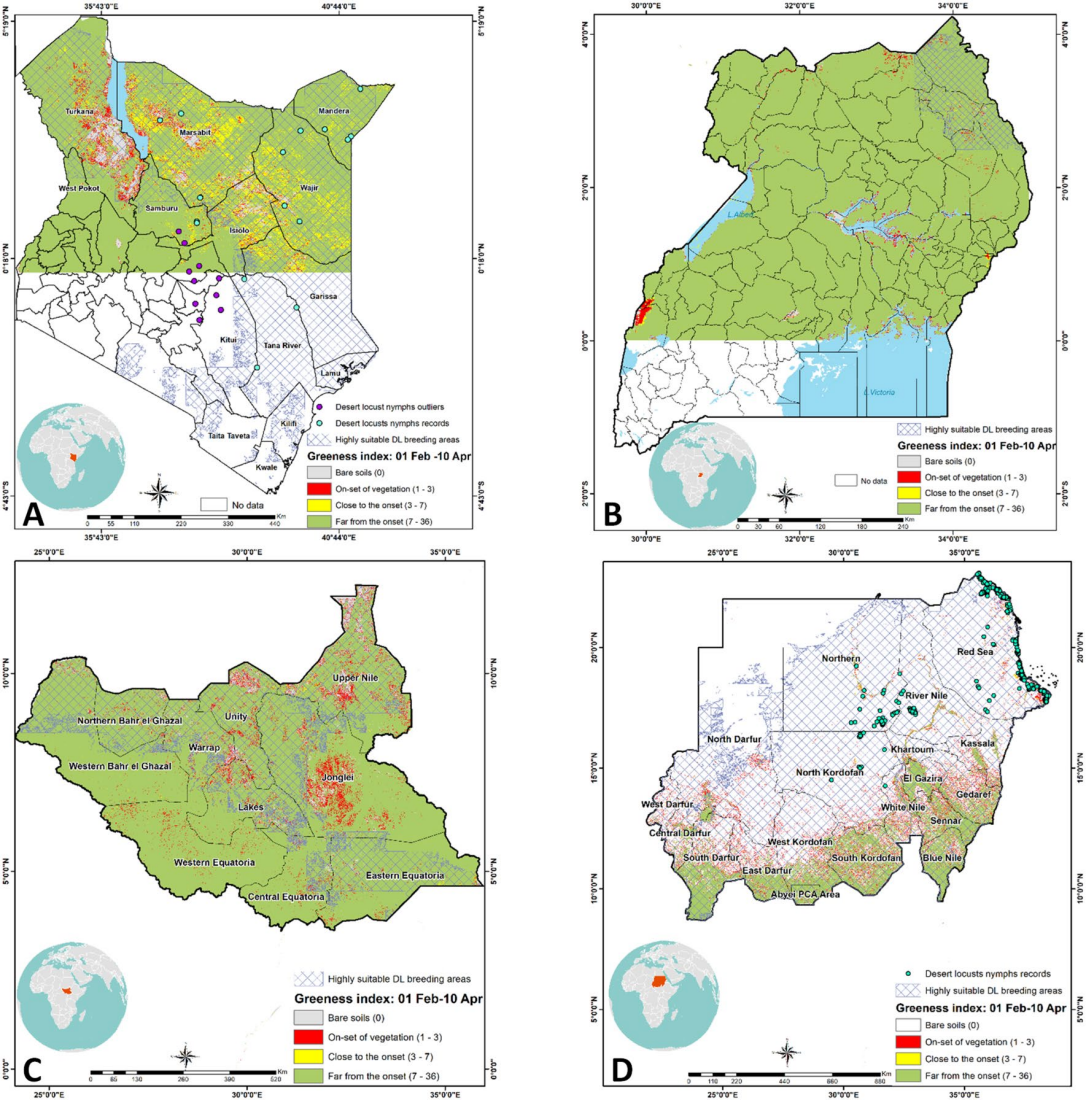
Overlaid layer of desert locust habitat suitability area (> 0.6 probability) with the greenness index from February–April 2020: (**A**) Kenya, (**B**) Uganda, (**C**) South Sudan, and (D) Sudan. Values ranging from 1–3 shaded in red indicate the areas with on-set of vegetation favorable for desert locust. Point locations with green vegetation that overlap with models’ outputs are actual desert locust highly suitable breeding areas.The figure was generated using the QGIS 3.10.2 software (https://qgis.org/downloads/)^21^.

Based on the overlaying of desert locust habitat suitability area (> 0.5 probability) with the vegetation layer (Fig. 6) of these countries, it is predicted that the likelihood for desert locust breeding is high in northeastern Kenya (Mandera, Wajir, Marsabit, Garissa counties), Turkana, and a few sites in Samburu with the presence of edible vegetation and high suitability for desert locust breeding (Fig. 6A). Similarly, in Uganda, the likelihood of desert locust establishing a breeding population is high in Kaabong, Kotido, and Moroto, also with relatively lower vegetation density (Fig. 6B). In South Sudan, Unity, Upper Nile, Eastern of the Equator, and some parts of Lakes and Jonglei are modeled to be suitable for the establishment of breeding populations (Fig. 6C). These regions are covered by low to moderately dense vegetation. However, in Sudan, most of the breeding populations occurred in regions with low to very low vegetation (mostly desert) (Fig. 6D).

**Figure 7:**
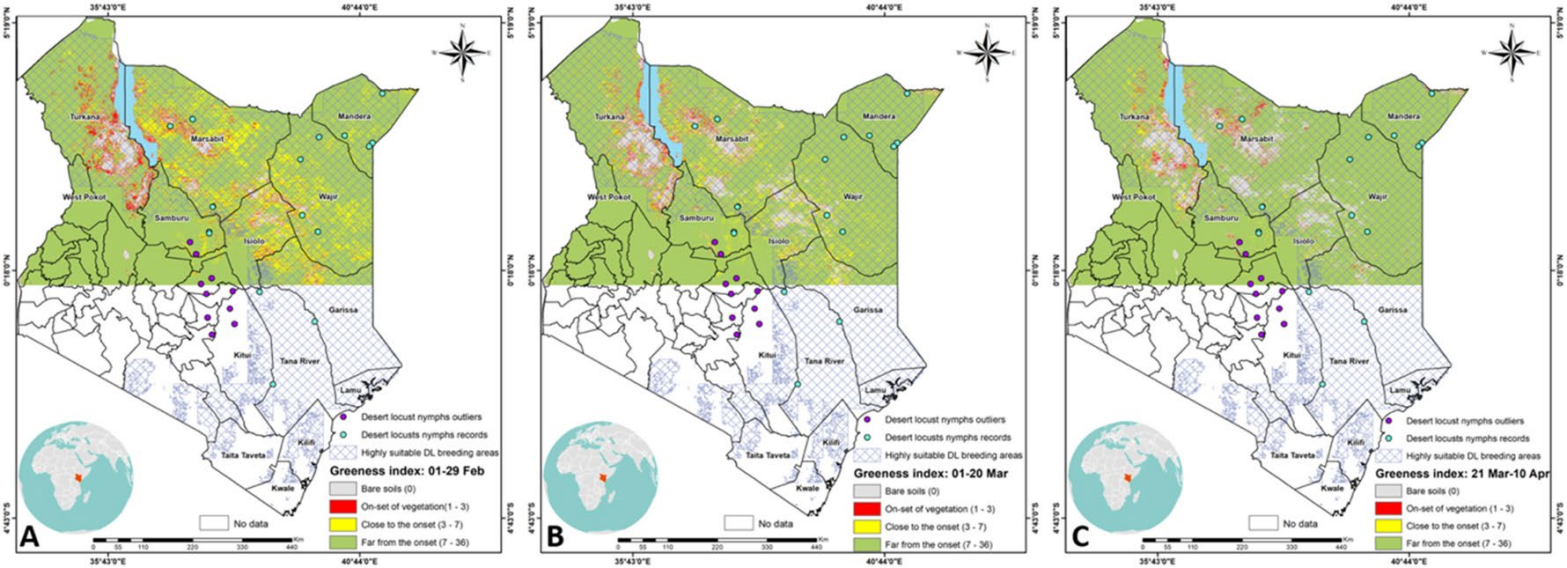
Temporal vegetation change from 1st Feb to 10th April (left to right) showing areas with on-set of vegetation favorable for desert locust breeding. These maps have shown that the potential location of desert locust breeding sites evolves with time in a predicted area in Kenya. The figure was generated using the QGIS 3.10.2 software (https://qgis.org/downloads/)^21^.

Within the potential desert locust breeding areas, the temporal vegetation change from 1st Feb to 10th April highlight particular sites favorable for desert locust breeding and illustrate how these sites evolve with time in Kenya (Fig. 7). The maps indicate the temporal vegetation change transitioning from one month to the next. The regions shaded in red indicate the on-set of vegetation.

## Discussion

All datasets used in the modeling experiments were obtained from secondary sources; no survey for ground scouting was conducted. The study, therefore, aligns with the concept promoted by the open science movement that is encouraging the reuse of data for further discovery and advisory^21^. Models that use only presence data are easier to develop and are popular as opposed to those that use input presence and absence data conjointly^20^. Presence data are usually simpler to obtain while confirming the absence of an organism involves extensive and detailed surveys^18,19^. However, in the absence of reliable data, models using both presence and absence datasets should be preferred^22^. But, a number of factors must be met. First, observed presence records used in the modeling exercises should be the outcome of a well-structured random sampling, which minimizes bias. Second, the occurrence records during sampling should not vary with any covariate that determines the suitability probability^22^. Given these challenges we applied a practical and robust modeling framework to predict the desert locust ecological breeding niche using existing presence data and ecological variables of three countries- Morocco, Mauritania, and Saudi Arabia, followed by projections to these countries. The best model was used to estimate the potential suitability to other countries, including Kenya, Uganda, South Sudan, and Sudan. Our assumptions are further substantiated by the fact that presence records collected within a country most likely follow a similar protocol, and lumping multiple datasets from many countries with different sampling protocols may provide a bias in the output of the assessment. Similarly, Piou and co-authors^23^ used historical desert locust occurrence observations to model the spatiotemporal distribution of desert locust in Mauritania and Morocco and found that it was possible to estimate the probabilities of seasonal desert locust breeding area.

We used an innovative model validation protocol in a such way that the MaxEnt machine learning algorithm was employed with a cross-validation strategy to avoid overfitting^24,25^. This technique consists of splitting the available records into two sets; the training set representing approximately 70% and the test data corresponding to 30% of the records. After the split, the test set is kept aside, and 40% to 50% is randomly extracted from the training dataset and used to run and lean MaxEnt model interactively. The validation exercise uses the remaining training datasets. The model development ends with the evaluation process that uses the test data. In addition to the cross-validation, we modified the strategy by using 100% of the presence records in the country, and we introduced another approach to evaluate the model. The model developed with data originating from country A (Morocco) was evaluated with a complete independent (not used for the training experiment) record from country B (Mauritania) and country C (Saudi Arabia) and vice versa. Only the model that performed well against different datasets from the different regions was reasonably used to create predictions for the targeted countries viz., Kenya, Uganda, Southern Sudan, and Sudan. To make the process more robust, we fitted known geo-referenced data from two projected countries (Kenya and Sudan) to measure and confirm the ability of the developed model to predict the breeding sites successfully.

We used environmental variables to characterize the probability of the desert locust breeding sites suitability, and all models developed provided an identical ranking of the percentage contribution of these variables but with differences in the level of contribution for each variable. This discrepancy may be explained by the quantity of data used as a proportion of the total size area of the country. MaxEnt, as most learning algorithms, is a data-driven machine; the more representative the data is, the more optimized is the learning process and the accuracy of the prediction^18,19^.

Surface temperature emerged as a critical factor, followed by soil moisture and sand content. Female desert locusts are known to prefer warmer and more open sites for initiating probing and digging activity for oviposition^26^. After the selection of sites based on surface temperature, females chose oviposition sites based on soil parameters, such as dry, soft, and sandy surface soil. The soil moisture at surface up to 6 cm is not important for selection, but at depths beyond 6 cm, soil moisture is critical for selection^2,26^. Rainfall emerged as the least important variable for desert locust breeding suitability. However, Adu-Acheampong and co-authors^15^ revealed that desert locust eggs could remain undeveloped for years but start to hatch with the onset of rainfall. This is perhaps because rainfall contribution is somehow already captured by the soil moisture.

Predicting the potential sites where desert locust can breed is of paramount importance. The current study provides information for preparedness and prioritization of ground surveillance on desert locust breeding and deployment of best-bet solutions for effective management of desert locust. The outcome of the study will assist policy makers in prioritizing resource allocation and management actions, such as targeted surveillance, the establishment of monitoring networks, mobilization of locust control products, and their application. The present study demonstrated that vast areas in Kenya are at high risk of becoming home to these pests. Currently, the swarms have stretched to over 17 counties reaching area south of equator like Embu. Our findings did not identify this location (Embu) as a potential breeding site. However, despite extensive swarming locust oviposition happens only in the most suitable habitats as observed earlier in the 1950s when, Kenya witnessed the last major locust upsurge^12^. The hatching and survival of eggs will heavily depend on the prevailing environmental conditions that should be similar to what prevails in the northern region of Kenya. Adult desert locust eggs laid in areas such as Mandera, Wajir, Garissa, Marsabit, Turkana, and few sites in Samburu can survive and hatch within weeks or remain undeveloped for years and, as soon as the environmental conditions become favorable, the cycle will continue due to the species fast-changing behavior. With the magnitude of the ongoing desert locust invasion in Kenya, it may become necessary to establish a permanent monitoring unit within the country. The maps generated in the present study could guide such units to undertake focused and cost-effective monitoring efforts. In Uganda, few sites in the north eastern regions have a high potential for supporting breeding populations. Based on the area coverage of the desert locust breeding suitability in the country, it may not be necessary to invest much for constant monitoring. However, Uganda may establish a task force that will work with Kenya's monitoring team for anticipated actions in case of possible outbreak conjointly. Suitability for desert locust establishing a breeding population is high in the south eastern and northern regions of South Sudan.

To improve the model predictions, vegetation, considered as a catalyst for desert locust selecting breeding locations, was overlaid onto the projected maps in targeted countries. Previous reports have indicated that type of vegetation is critical for desert locust oviposition and breeding. Ecotone belts and mosaic vegetation are more preferred over uniform vegetation and topography for females to settle and oviposit^25^. Although we noted some gaps in the location of green vegetation for the southern part of Kenya and Uganda, this study provides a reasonable tool that will guide survey teams to monitor potential breeding areas and avoid unnecessary guesses and investment for monitoring. However, detailed assessment of the temporal variations in prevalence of vegetation and desert locust breeding, type of vegetation, and other metrics and inclusion of such metrics in the modeling could aid in fine-tuning the model and adding precision to the prediction. Beyond the prediction of breeding sites, the current desert locust outbreak is triggered by a change in rainfall pattern which expands areas of potential invasion as a consequence of climate change, and other marginally suitable areas and conditions may become suitable in the nearest future hence the need to monitor and generate data for refining the current model continuously.

## Methodology

### Global data compilation and developing prediction models for desert locust breeding sites

**Figure 8:**
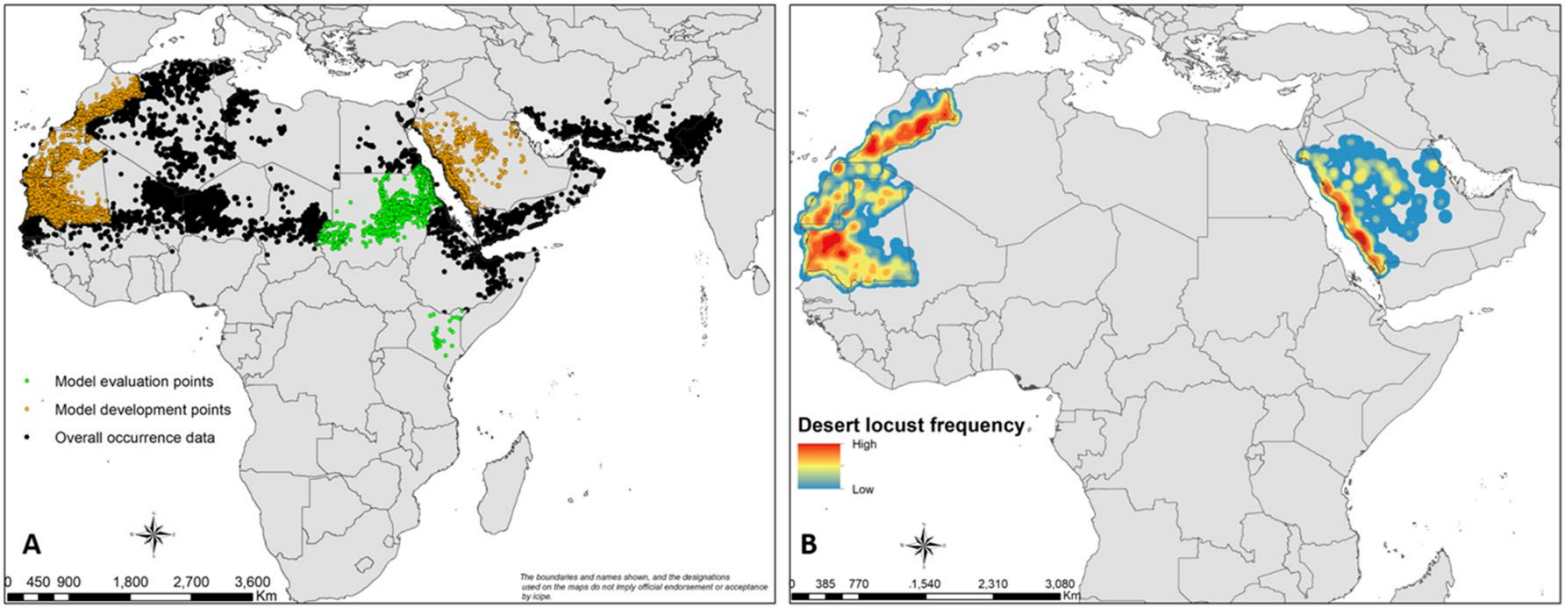
(**A**) Desert locust breeding sites collected in Africa, the Middle East, and Western Asia from 1985 to 2020. The brown dots are the records used for developing and validating the model; the green dots represent the breeding sites used for measuring the model performance, while the black dots are known breeding sites for desert locust. (**B**) Heat map indicating the hotspot areas where the majority of desert locust nymph presence records were collected from 1985 to 2020 in Mauritania, Morocco, and Saudi Arabia. High intensity of desert locust nymph presence is characterized by red color, while the blue color displays a low repetitive occurrence in the same point location of desert locust nymph. The figure was generated using the QGIS 3.10.2 software (https://qgis.org/downloads/)^21^.

A search for information related to the occurrence/incidence of desert locust breeding sites was carried out through Google and Web of Science. The keywords used for the search comprised the following: desert locust breeding sites, desert locust band locations, hopper molt locations, and desert locust swarm sites. The focus was given to countries in which the government invests significant resources on desert locust control, such as Morocco. On the Moroccan anti-desert locust centre website (https://www.criquet-maroc.ma), a monthly report of the presence of desert locust at different stages is provided. Extensive time-series data were obtained from the Food and Agriculture Organization (FAO) of the United Nations. Further data were also obtained from survey reports and archives from the FAO website (https://www.fao.org/ag/locusts/en/info/info/index.html). Although we accessed a total of 9,134 records, the development of the model only used 5,406 breeding sites from Morocco (367), Mauritania (2,661) and Saudi Arabia (1,703) (Fig. 8A) dating from 1985–2020. Data for measuring the performance of the developed model for Kenya (n = 28) and Sudan (n = 647) were obtained from the Plant Protection Services from the Ministry of Agriculture, Livestock and Fisheries, the Desert Locust Control Organization (DLCO) and FAO.

### Assumptions and pre-processing of data

The study assumed that all records were randomly obtained from a larger area within the country, as described in the FAO standard operating procedure (SOP) for desert locust field surveys^2^. Although countries are directed to use the FAO SOP for surveys, the accuracy of the sample size and quality and manipulation of the data may vary between countries. Therefore, we have considered a country as a unit of metric and did not lump records obtained from distinct countries for developing the models. For each country, environmental predictor variables were organized so that projections, grid cell sizes, and their alignment, and spatial coverage were regular across all layers. The overall datasets (Fig. 8A) have many records per grid cell because of either repeated occurrence over the years or sites being near to each other. Therefore, we identified hotspots and clustering presence record points for a selected country for the modelling exercise. From this analysis, a heat map was obtained (Fig. 8B) to highlight the frequency of desert locust breeding activity. This map resulted from the kernel density estimation tool using the open source QGIS 3.10.2 software (https://qgis.osgeo.org)^20^. The density is calculated based on the number of points in a location, with larger numbers of clustered points resulting in high intensity. This heat map provided guidance in the choice of countries used for model development. Further, it substantiated the selection of the best model used for the projections in countries of East Africa that did not have breeding site records for the past 25 years.

### Bio-climatic and edaphic variables for modelling desert locust breeding suitability areas

Temperature, rainfall, soil moisture, and sand content are the most critical environmental variables for desert locust breeding locations^14^. Data on these variables were sourced from different platforms; monthly long term (1970–2000) average temperature and rainfall datasets were sourced from Worldclim2 data platform (https://worldclim.org/)^27^, long term (1948–2020) average soil moisture was sourced from National Oceanic and Atmospheric Administration (NOAA) Climate Prediction Center (https://www.psl.noaa.gov/data/gridded/data.cpcsoil.html)^28^ and sand content for 2016 at a depth of 5–15 cm was downloaded from the International Soil Reference and Information Centre (ISRIC) Data Hub (https://data.isric.org)^23^. For temperature and rainfall, an average of four months (December, January, February, and March) for each variable was calculated and used in the model. These four months correspond to the desert locust invasion in East Africa. Rationally, the environmental variables used for our modelling experiments should be of identical years with the desert locust occurrence data. These data mismatch motivated the choice of our methodology. Machine learning has been reported to not be limited to prediction only, but the algorithms like MaxEnt has the potential to improve the efficiency and effectiveness of the modelling experiments by correcting and overcoming data gaps especially in the context of long term time series^29^. All datasets were pre-processed and adjusted to a uniform spatial resolution of 1 km before the modeling experiments.

### Modelling desert locust breeding suitability

Presence records of desert locust breeding locations in Mauritania, Morocco, and Saudi Arabia were pooled with environmental data using the MaxEnt^17^ machine learning algorithm to estimate the suitable areas for oviposition at local and regional scales. MaxEnt performs relatively well in the context of developing a model using presence records only^14^. The algorithm predictions are logistic and based on the ability to estimate a distribution of probability based on the physics principle of maximum entropy, which satisfies a set of checks from environmental variables^24^. In the context of this study, we assumed that all data points from each country that were used in the modeling were obtained through laborious sampling protocols that include selection bias. Hence, we consider the MaxEnt output as the level of environmental suitability indicating a desert locust ecological breeding niche, expressed in probability.

Practically, the modeling experiments were carried out in multiple steps. First, MaxEnt was ran sequentially using presence records from Mauritania, Morocco, and Saudi Arabia to predict the suitable habitat sites for desert locust breeding in these countries. For each country, the model output was overlaid to its presence record layer surfaces serving as a channel to extract the probability value of the pixel at each desert locust breeding point. The obtained probability values were then used to estimate the model goodness-of-fit using the area under the curve (AUC) of the receiver operating characteristic curve (ROC). Secondly, the model developed in one country was projected in the other two countries to test for goodness-of-fit. Specifically, the model developed from Morocco presence records was projected to Saudi Arabia and Mauritania. Similarly, the model developed with Saudi Arabia's presence records was projected to Morocco and Mauritania. Finally, the model we developed with Mauritania's presence records was projected to Morocco and Saudi Arabia. For all projections, desert locust occurrence points were overlaid on the projected layer surfaces and then used to extract the probability values of the pixel where each point lies in the projected raster layer. The analysis generated breeding area maps for Saudi Arabia and Mauritania from Morocco model projections. The Morocco and Mauritania breeding area maps developed from Saudi Arabia model projections. Likewise, the Morocco and Saudi Arabia maps obtained from Mauritania model projections. To control overfitting, the individual run was repeated three times using the cross-validation approach, and the results presented in this study are products of an ensemble of the three modeling experiments. The obtained maps were compared with known presence point locations in the projected countries using a histogram and a normal distribution fitting curve. Descriptive statistics were also generated from the model outputs to recognize which among the projected models better captured the presence records in the country of projection. We only present the descriptive statistic results of the best model projecting the desert locust breeding sites to the other countries. Acknowledging that statistics alone cannot provide satisfaction of the ecological rationality of the model outputs, we confirmed the results with a visual lens.

### Projection of the best fit model to predict desert locust breeding sites

The best model parameters among the three countries (i.e., Morocco, Saudi Arabia, and Mauritania), were then used to project breeding suitability in Kenya, Uganda, South Sudan, and Sudan. After modeling the desert locust breeding suitability, we performed a comparison of the level of matching of the projected desert locust breeding suitability with geo-referenced points with known desert locust breeding activity from field surveys in Kenya and Sudan. The purpose of this exercise was to measure the model performance and confirm its ability to predict suitable breeding sites in targeted regions accurately.

### Vegetation impact on desert locust breeding sites

A product developed by Vlaamse instelling voor technologisch onderzoek (VITO)^12^ to monitor temporal changes in vegetation over the desert locust recession and invasion areas were sourced to assess the impact of vegetation on the breeding sites in Kenya, Uganda, South Sudan, and Sudan. The product combines daily observations from SPOT and MODIS satellite imagery using the middle infrared, near-infrared and red bands to develop greenness estimates^12^. The product is developed every ten days at a resolution of 250 m. The index is provided as a time meter, indicating the number of dekades since the vegetation on-set^12^. This highlights the number of dekades a pixel appears to have vegetation from its on-set to the current dekade; hence the greenness is a measure of the transition from no vegetation to the startup of vegetation occurrence^12^. This helps in detecting vegetation emergence in arid and semi-arid regions. The 10-days dynamic maps dating from the beginning of February 2020 to the first week of April 2020 were downloaded from the VITO website, analyzed, and overlaid in the developed model outputs. This is used to dynamically detect the progression of the changes of breeding sites locations due to the temporal variation of the on-set of vegetation in East Africa. Values from the model indicating high probability (> 0.5) of habitat suitability of desert locust in Kenya, Uganda, South Sudan, and Sudan were masked out and overlaid on the vegetation layer to assess the relationship between vegetation and breeding sites for the desert locust**.**
